# Aryl hydrocarbon receptor regulates IL-22 receptor expression on thymic epithelial cell and accelerates thymus regeneration

**DOI:** 10.1038/s41536-023-00339-7

**Published:** 2023-11-08

**Authors:** Jingyi Shen, Ying Wang, Fei Zheng, Shuo Cao, Qiu Lan, Kailin Xu, Bin Pan

**Affiliations:** 1 Blood Diseases Institute, Xuzhou Medical University, Xuzhou, 221002 China; 2 Department of Hematology, The Affiliated Hospital of Xuzhou Medical University, Xuzhou Medical University, Xuzhou, 221002 China

**Keywords:** Allotransplantation, Graft-versus-host disease

## Abstract

Improving regeneration of damaged thymus is important for reconstituting T-cell immunity. Interleukin-22 (IL-22) was proved to improve thymus regeneration through recovering thymic epithelial cells (TECs). The IL-22 receptor IL-22RA1 is crucial for mediating IL-22 functions. Mechanism that regulates IL-22RA1 expression is unknown. Through using TECs-conditional knockout mice, we found aryl hydrocarbon receptor (AHR) is important for thymus regeneration, because Foxn1-cre-mediated AHR knockout (*Ahr*KO) significantly blocks recovery of thymus cells. Giving mice the AHR inhibitor CH-223191 or the AHR agonist FICZ blocks or accelerates thymus regeneration, respectively. *Ahr*KO-mediated blockade of thymus regeneration could not be rescued by giving exogenous IL-22. Mechanistically, *Ahr*KO mice shows decreased IL-22RA1 expression. In the murine TECs cell line mTEC1 cells, targeting AHR shows an impact on IL-22RA1 mRNA levels. Using chromatin immunoprecipitation and luciferase reporter assays, we find AHR co-operates with STAT3, binds the promotor region of IL-22RA1 gene and transcriptionally increases IL-22RA1 expression in mTEC1 cells. Foxn1-cre-mediated IL-22RA1 knockout (*Il22ra1*KO) blocks thymus regeneration after irradiation. Furthermore, targeting AHR or IL-22RA1 has significant impacts on severity of murine chronic graft-versus-host disease (cGVHD), which is an autoimmune-like complication following allogeneic hematopoietic cell transplantation. Giving FICZ decreases cGVHD, whereas *Il22ra1*KO exacerbates cGVHD. The impacts on cGVHD are associated with thymus regeneration and T-cell immune reconstitution. In conclusion, we report an unrecognized function of TECs-expressed AHR in thymus regeneration and AHR transcriptionally regulates IL-22RA1 expression, which have implications for improving thymus regeneration and controlling cGVHD.

## Introduction

Thymus is a central organ in vertebrates for generating a functionally competent T-cell immunity which protects the body against foreign antigens and maintains self-tolerance^1^. However, thymus is sensitive to stresses caused by external environment changes and insults such as infections and cytotoxic treatment^2^. Thymus shows an ability of regeneration after damage, but the ability decreased with aging and in some extreme settings such as allogeneic hematopoietic cell transplantation (allo-HCT)^3^. Strategies to improve thymus regeneration will protect older humans from infections and accelerate reconstitution of T-cell immunity of allotransplant patients^4,5^.

Thymic epithelial cells (TECs) compose the major part of the microenvironment, which supports thymocytes development. Under the nursing of TECs, thymocytes proliferate and acquire self-tolerance^6^. TECs could be injured by damages, such as irradiation and chemotherapies. Recovery of TECs is fundamental for reconstitution of T-cell immunity^7^. Interleukin-22 (IL-22) was proved to accelerate thymus regeneration and restore thymus function through recovering TECs^8,9^. IL-22 is produced by immune cells including T cells and innate lymphoid cells, whereas the receptors for IL-22 are expressed by epithelial cells, an interesting cross-talk between immune cells and nonimmune cells^10^. We previously found IL-22 improved thymus regeneration through activating a STAT3/Mcl-1 pathway^9^, indicating the IL-22 receptor IL-22RA1 is important in mediating the effect of IL-22 because IL-22RA1 transduces signals through STAT3^10^. IL-22 shows a significant pro-regenerative function in thymus but is still not fully effective^9^. It is unknown whether IL-22RA1 is indispensable for thymus regeneration. The mechanism that regulates IL-22RA1 expression is also poorly understood.

Aryl hydrocarbon receptor (AHR) is a critical transcription factor that regulates IL-22 expression in T cells and innate lymphoid cells^10,11^. Given that intrathymic T-cell-derived IL-22 improved thymus regeneration^12^, we found T-cell-specific knockout of AHR impaired thymus regeneration which could be reversed by giving exogenous IL-22 in our preliminary study. However, giving exogenous IL-22 failed to accelerate thymus regeneration in irradiated mice treated by the AHR inhibitor CH-223191. These preliminary results indicated AHR also had an impact on other factors that regulated thymus regeneration. In this study, we found AHR transcriptionally increased IL-22RA1 expression of TECs. Both AHR and IL-22RA1 in TECs were important for thymus regeneration. Giving mice the AHR agonist FICZ improved thymus regeneration. Targeting AHR or IL-22RA1 had a significant impact on the pathogenesis of murine chronic graft-versus-host disease (cGVHD), an autoimmune-like complication following allo-HCT^13^.

## Results

### AHR regulated thymus regeneration after irradiation-induced injury

**Fig. 1:**
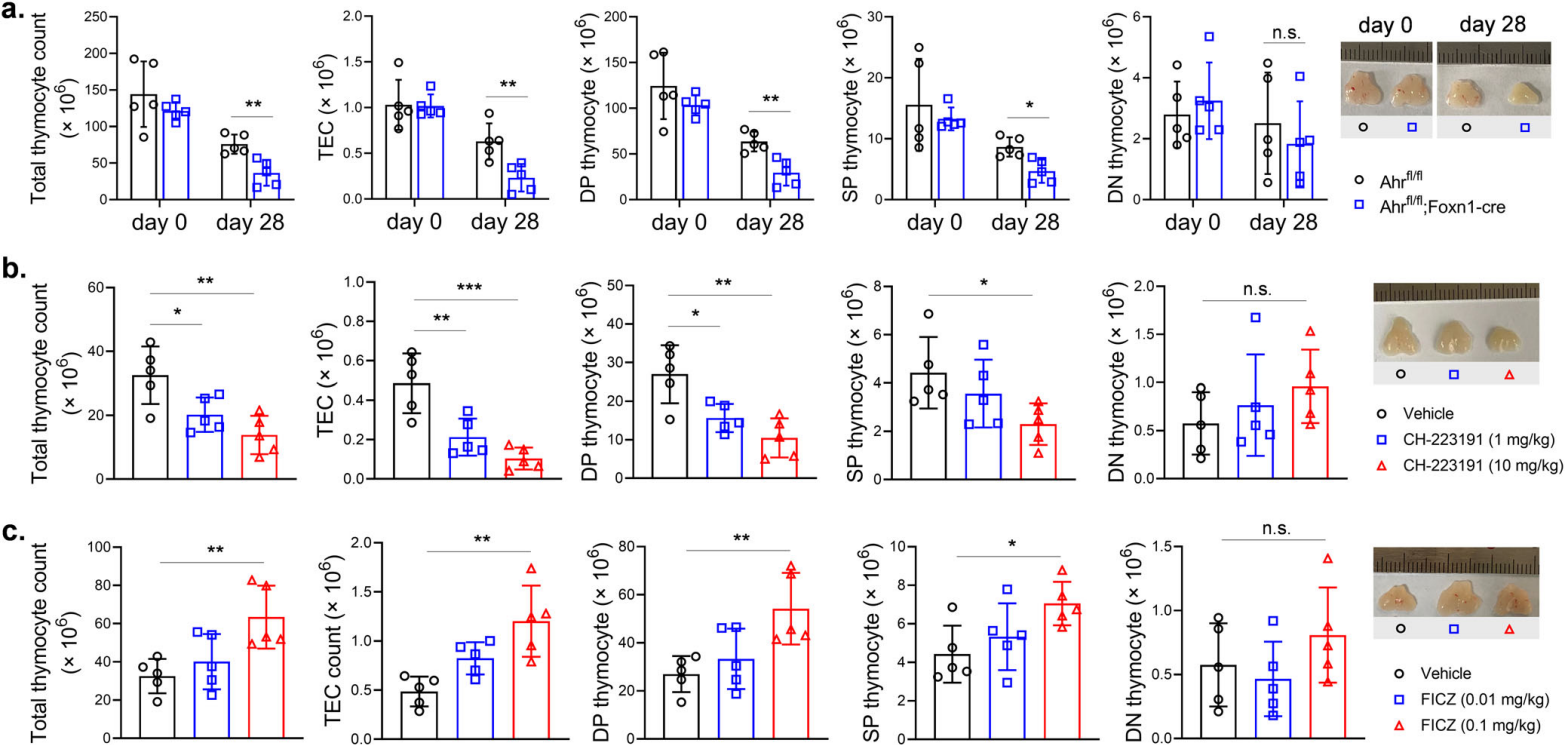
AHR regulated thymus regeneration after irradiation-induced injury. **a** B6-*Ahr*^*fl/fl*^ mice and B6-*Ahr*^*fl/fl*^*;Foxn1-cre* mice were treated by 5.5 Gy TBI. At days 0 and 28, thymii were collected and cells were dissected by digestion. Total thymus cells were counted. TECs and thymocyte subpopulations were counted according to total cell count and proportions from flow cytometric analysis (*n* = 5 in each group). Day 0 indicates the samples were collected right before TBI. **b**, **c** Wild-type C57BL/6 mice, pretreated by 5.5 Gy TBI, were intraperitoneally injected with vehicle, CH-223191 or FICZ thrice weekly for 2 weeks. At day 14, thymus cells were counted as described (*n* = 5). Data are mean ± SD, compared using one-way ANOVA test or Student’s *t* test. *, *p* < 0.05; **, *p* < 0.01; ***, *p* < 0.001; n.s., not significant.

To determine whether TECs-expressed AHR played a role in thymus regeneration, we generated Foxn1-cre-mediated AHR knockout (*Ahr*KO) mice because Foxn1 is preferentially expressed in TECs. The littermates without Foxn1-cre expression were used as controls. We treated the mice with sublethal dose of TBI and let them to recover without any treatment. At days 0 and 28, thymi were collected and cells were dissected by digestion. TECs and thymocytes were counted according to total cell count and flow cytometric analysis (Supplementary Figs 1 and 2). Day 0 indicates the samples were collected right before TBI. The counts of intrathymic cells were comparable in control mice and *Ahr*KO mice at day 0. Whereas, at day 28, *Ahr*KO mice had lower cell counts of total thymocytes, DP thymocytes, SP thymocytes and TECs comparing with controls. No difference was observed regarding the counts of DN thymocytes in the two groups at day 28. The photos indicated sizes of thymii were consistent with cell counts (Fig. 1a).

Given that TECs-expressed AHR was important for the self-regenerative function of the thymus, we interrogated whether the pharmacologic intervention of AHR would have an impact on thymus regeneration. We applied CH-223191 and FICZ, a well-documented AHR inhibitor and an AHR agonist, to treat sublethally irradiated wild-type mice. The inhibitor CH-223191 decreased the size and total cell count of thymii at the dose of 1 mg/kg and 10 mg/kg. CH-223191 also decreased counts of DP thymocytes, SP thymocytes and TECs. The effect was positively related to dosage (Fig. 1b). On the contrary, the agonist FICZ increased thymus size and counts of intrathymic cells at the dose of 0.1 mg/kg (Fig. 1c). DN thymocytes counts were not altered by CH-223191 or FICZ (Fig. 1b, c).

### Knockout of AHR in TECs decreased IL-22RA1 expression

**Fig. 2:**
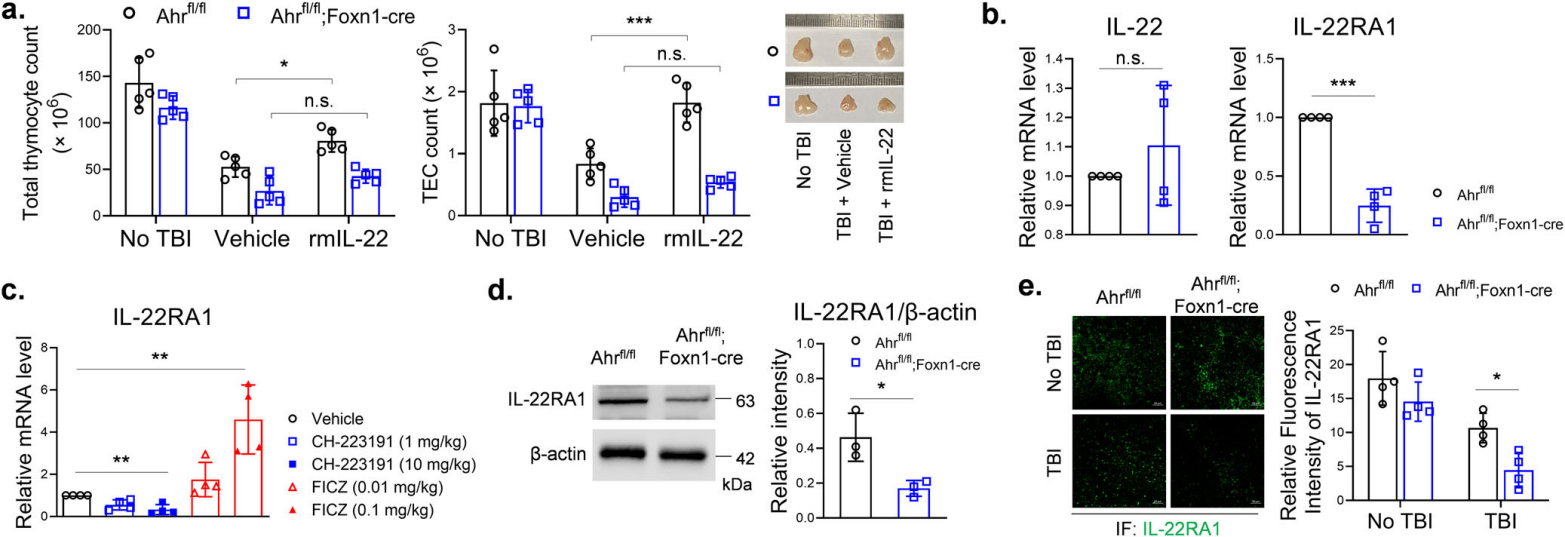
Knockout of AHR in TECs decreased IL-22RA1 expression. **a** B6-*Ahr*^*fl/fl*^ mice and B6-*Ahr*^*fl/fl*^*;Foxn1-cre* mice, pretreated by 5.5 Gy TBI, were intraperitoneally injected with vehicle or rmIL-22 (200 ng) thrice weekly for 2 weeks. At day 14, thymus cells were counted as described (*n* = 5). **b**, **c** Thymii were collected from irradiated mice. qPCR was performed to detect mRNA levels of IL-22 and IL-22RA1 (*n* = 4). The values of control groups were set as 1, and the values of other groups were 2^–ΔΔCT^ relative to controls. **d** Thymic stromal cells were isolated from irradiated mice (*n* = 3). Western blot was performed with indicated antibodies. Band intensity was analyzed using ImageJ. **e** Cryosections of thymii were stained by immunofluorescence with anti-IL-22RA1 (*n* = 4). Fluorescence intensity was analyzed using ImageJ. Scale bar: 50 μm. Data are mean ± SD, compared using one-way ANOVA test or Student’s *t* test. *, *p* < 0.05; **, *p* < 0.01; ***, *p* < 0.001; n.s., not significant.

Because AHR regulates the expression of IL-22^11^, we gave irradiated *Ahr*KO mice rmIL-22 to test if this would reverse the effect of Foxn1-cre-mediated AHR knockout. Giving rmIL-22 increased cell counts of total thymocytes and TECs in control mice, but failed to increase those cell counts in *Ahr*KO mice. The result of cell count was supported by thymus size (Fig. 2a). Interestingly, Foxn1-cre-mediated AHR knockout did not alter IL-22 mRNA level but decreased IL-22RA1 mRNA level in the thymii of irradiated mice (Fig. 2b). In irradiated wild type mice, CH-223191 and FICZ treatments decreased and increased intrathymic IL-22RA1 mRNA level, respectively (Fig. 2c). We isolated thymic stromal cells from irradiated mice, and found *Ahr*KO decreased IL-22RA1 protein levels (Fig. 2d). Immunofluorescence staining also showed *Ahr*KO decreased IL-22RA1 expression in thymus slides of irradiated mice (Fig. 2e). These results indicated AHR might have a potential in regulating intrathymic IL-22RA1 expression, which provide a basic for IL-22’s action.

### AHR transcriptionally increased IL-22RA1 expression in TECs

**Fig. 3:**
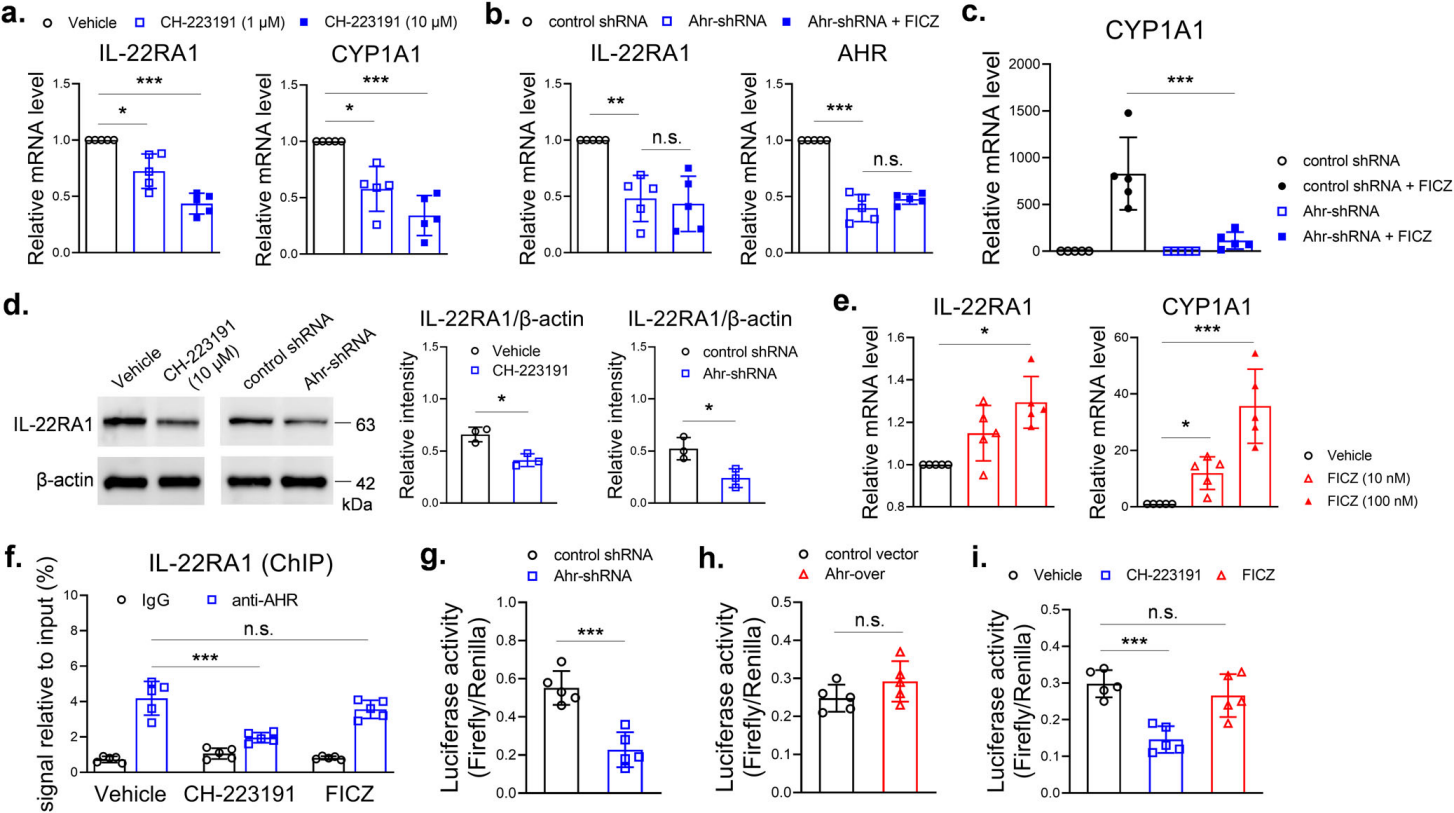
Targeting AHR had an impact on IL-22RA1 expression in mTEC1 cells. **a** CH-223191 was used to treat mTEC1 cells for 24 h. **b**, **c** mTEC1 cells, stably transduced with control shRNA or Ahr-shRNA lentivirus, were treated with vehicle or FICZ (100 nM) for 24 h. **d** Total cell proteins were extracted from the mTEC1 cells as described above. Western blot was performed with indicated antibodies (*n* = 3). **e** FICZ was used to treat mTEC1 cells for 24 h. qPCR was performed to detect mRNA levels in cells as described (*n* = 5). **f** mTEC1 cells were treated by vehicle, CH-223191 (10 μM) or FICZ (100 nM) for 24 h. ChIP assay was performed with anti-AHR. The precipitated DNA was detected by qPCR with primers of mouse IL-22RA1 gene promotor (*n* = 5). **g** mTEC1 cells stably transduced with control shRNA or Ahr-shRNA lentivirus, **h** mTEC1 cells stably transduced with control or Ahr-overexpression lentivirus, **i** mTEC1 cells, without genetic modification, were treated by vehicle, CH-223191 (10 μM) or FICZ (100 nM) for 24 h. **g**, **h**, **i** These cells were transfected with pGL4.10 plasmids (firefly, following mouse IL-22RA1 gene promoter sequence) and pRL-TK plasmids (renilla, normalizer) for 12 h. Luciferase activity was measured (*n* = 5). Data are mean ± SD, compared using one-way ANOVA test or Student’s *t* test. *, *p* < 0.05; **, *p* < 0.01; ***, *p* < 0.001; n.s., not significant.

Next, we used the murine TECs cell line mTEC1 to test the direct action of AHR in TECs. The inhibitor CH-223191 decreased mRNA levels of IL-22RA1 and CYP1A1 in mTEC1 cells (Fig. 3a). CYP1A1 is a reporter gene of AHR pathway activity and was used here as a positive control^11^. We also used shRNA to knock down AHR expression. Knockdown of AHR decreased IL-22RA1 mRNA level, an effect not reversed by treatment with the agonist FICZ (Fig. 3b). FICZ dramatically increased CYP1A1 mRNA level in control shRNA treated mTEC1 cells, and this effect was significantly inhibited by knockdown of AHR (Fig. 3c). Western blot analysis showed both CH-223191 and Ahr-shRNA decreased protein levels of IL-22RA1 in mTEC1 cells (Fig. 3d). FICZ increased IL-22RA1 mRNA level to 1.3 times as compared with control in mTEC1 cells, an effect not as significant as that in CYP1A1 mRNA level (Fig. 3e).

**Fig. 4:**
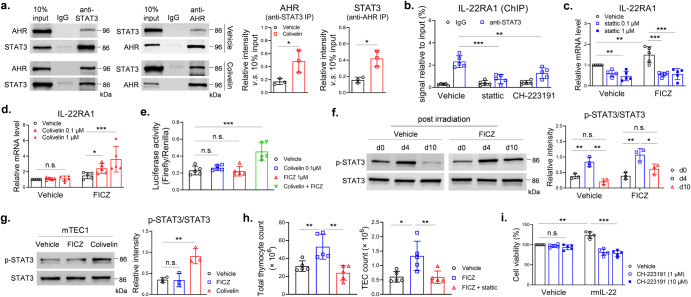
AHR transcriptionally increased IL-22RA1 expression in mTEC1 cells. **a** mTEC1 cells were treated by vehicle or colivelin (1 μM) for 24 h. Immunoprecipitation was performed with anti-STAT3 or anti-AHR (*n* = 3). Western blot was used to detect proteins in the precipitate. **b** mTEC1 cells were treated by vehicle, stattic (1 μM) or CH-223191 (10 μM) for 24 h. ChIP assay was performed with anti-STAT3 as described (*n* = 5). **c**, **d** mTEC1 cells were treated by vehicle, stattic or colivelin for 24 h with/without FICZ (100 nM). qPCR was performed to detect mRNA levels in cells as described (*n* = 5). **e** mTEC1 cells were treated by colivelin and/or FICZ for 24 h (*n* = 5). Luciferase reporter assay was performed as described. **f**, **g** Total proteins were extracted from thymic stromal cells isolated from irradiated wild type mice which were treated by vehicle or FICZ (*n* = 3). Whole-cell proteins were extracted from mTEC1 cells treated by vehicle, FICZ (100 nM) or colivelin (1 μM) for 24 h (*n* = 3). Western blot was used to detect proteins as indicated. **h** Wild type C57BL/6 mice, pretreated by 5.5 Gy TBI, were intraperitoneally injected with vehicle, FICZ (0.1 mg/kg) or combined FICZ (0.1 mg/kg) and stattic (10 mg/kg) thrice weekly for 2 weeks. At day 14, thymus cells were counted as described (*n* = 5). **i** mTEC1 cells were treated by vehicle or rmIL-22 (20 ng/ml) in the presence of different doses of CH-223191 for 24 h (*n* = 5). Cell viability was detected by CCK-8 assay. Data are mean ± SD, compared using one-way ANOVA test or Student’s *t* test. *, *p* < 0.05; **, *p* < 0.01; ***, *p* < 0.001; n.s., not significant.

To test whether AHR regulates transcription of IL-22RA1 gene, we performed ChIP and luciferase reporter assays with mTEC1 cells. DNA fragments of IL-22RA1 gene promotor area (-1800bp–-1500bp of TSS) were detectable in anti-AHR immunoprecipitate from the ChIP assay, which indicated a direct binding of AHR and IL-22RA1 gene promotor area. This effect was inhibited by CH-223191, but not increased by FICZ (Fig. 3f). Ahr-shRNA reduced luciferase activity in mTEC1 cells that were transfected with firefly reporter vector containing the IL-22RA1 gene promotor area (-2000bp–-1300bp of TSS) (Fig. 3g), but over-expression of AHR did not increase luciferase activity in mTEC1 cells (Fig. 3h). Comparing with vehicle control, luciferase activity was reduced by CH-223191, whereas FICZ did not increase luciferase activity (Fig. 3i). These results suggested AHR was important for transcription of IL-22RA1 gene, however, activation of AHR alone did not significantly increase transcriptional activity of IL-22RA1 gene in mTEC1 cells. It was reported that STAT3 positively regulated IL-22RA1 expression^14^. We detected binding of STAT3 and AHR in mTEC1 cells, and the binding was enhanced by a STAT3 agonist colivelin (Fig. 4a). STAT3 antibody was used to perform ChIP assay. The IL-22RA1 gene promotor DNA fragments (-1800bp — -1500bp of TSS) were detectable in the immunoprecipitate, an effect inhibited by a STAT3 inhibitor stattic. The binding activity was also inhibited by CH-223191 (Fig. 4b). These pull-down assays indicated STAT3 and AHR bind to the same region of IL-22RA1 promotor. To test whether STAT3 contributes to IL-22RA1 expression, we used stattic and colivelin to treat mTEC1 cells. The STAT3 inhibitor stattic decreased mRNA levels of IL-22RA1 (Fig. 4c). The STAT3 agonist colivelin alone did not increased IL-22RA1 mRNA level, but increased IL-22RA1 mRNA level when concurrently used with FICZ (Fig. 4d). Luciferase reporter assay also showed concurrent use of colivelin and FICZ significantly increased transcription of IL-22RA1 gene (Fig. 4e). FICZ alone increased IL-22RA1 mRNA level to 1.3 – 1.5 times in mTEC1 cells (Figs. 3e and 4c), whereas the number was 4.5 times in irradiated thymus (Fig. 2c). Increasing the FICZ dose to 1 μM did not further increase IL-22RA1 mRNA level (data not shown). The efficacy of FICZ seems to be higher in increasing IL-22RA1 mRNA level in vivo. A possible reason was the different activation level of STAT3 between the in vivo and in vitro experimental settings. Phosphorylated STAT3 level increased in thymus stromal cells at day 4 in both control and FICZ-treated irradiated mice (Fig. 4f). In contrast, FICZ alone did not increase phosphorylated STAT3 level in the cultured mTEC1 cells (Fig. 4g), but colivelin increased phosphorylated STAT3 level and enhanced binding of STAT3 and AHR (Fig. 4a, g). We gave irradiated mice concurrent FICZ and stattic treatments. Stattic attenuated the effect of FICZ in promoting thymus regeneration after irradiation (Fig. 4h). We further showed inhibition of AHR abolished the proliferative effect of IL-22 in mTEC1 cells (Fig. 4i). Thus, AHR, probably co-operated with STAT3, regulated transcription of IL-22RA1 gene.

### IL-22RA1 was important for thymus regeneration after irradiation-induced injury

**Fig. 5:**
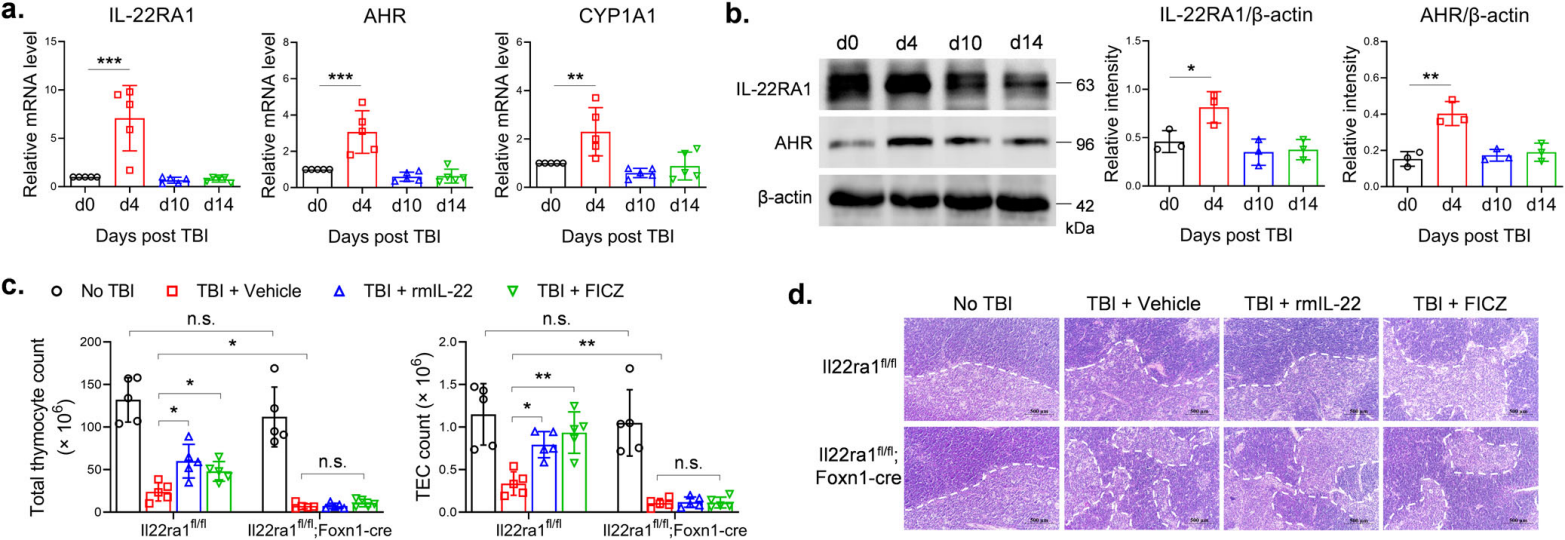
TECs-specific knockout of IL-22RA1 blocked thymus regeneration. **a**, **b** Thymic stromal cells were isolated from wild-type C57BL/6 mice treated by 5.5 Gy TBI. Day 0 indicates the samples were collected right before TBI. **a** qPCR was performed to detect mRNA levels (*n* = 5). The values of Day 0 were set as 1. **b** Western blot was performed with indicated antibodies. **c**, **d** B6-*Il22ra1*^*fl/fl*^ mice and B6-*Il22ra1*^*fl/fl*^*;Foxn1-cre* mice, pretreated by 5.5 Gy TBI, were intraperitoneally injected with vehicle, rmIL-22 (200 ng) or FICZ (0.1 mg/kg) thrice weekly for 2 weeks. At day 14, **c** thymus cells were counted as described (*n* = 5). **d** H&E staining was performed on slides of thymus. Scale bar: 500 μm. Data are mean ± SD, compared using one-way ANOVA test or Student’s *t* test. *, *p* < 0.05; **, *p* < 0.01; ***, *p* < 0.001; n.s., not significant.

We analyzed expressions of IL-22RA1, AHR and CYP1A1 in thymii of sublethally irradiated mice. The mRNA levels of IL-22RA1, AHR and CYP1A1 increased at day 4 after irradiation and decreased to baseline level at days 10 and 14 (Fig. 5a). Protein levels also showed similar changes (Fig. 5b). To confirm whether IL-22RA1 was important for thymus regeneration, we generated Foxn1-cre-mediated IL-22RA1 knockout (*Il22ra1*KO) mice. Without irradiation, there is no difference in the counts of total thymocytes and TECs between *Il22ra1*KO mice and controls. At day 14 after irradiation, *Il22ra1*KO mice had lower cell counts of total thymocytes and TECs comparing with controls. Treatment with rmIL-22 or FICZ increased cell counts of total thymocytes and TECs in control mice, but failed to do so in *Il22ra1*KO mice (Fig. 5c and Supplementary Fig. 3). Histological analysis showed the structure of thymic tissues changed after irradiation, especially the medulla shrank significantly in *Il22ra1*KO mice (Fig. 5d). These results indicated that IL-22- or FICZ-medicated regenerative effect depended on IL-22RA1 in TECs.

### Targeting AHR or IL-22RA1 had an impact on murine cGVHD

**Fig. 6:**
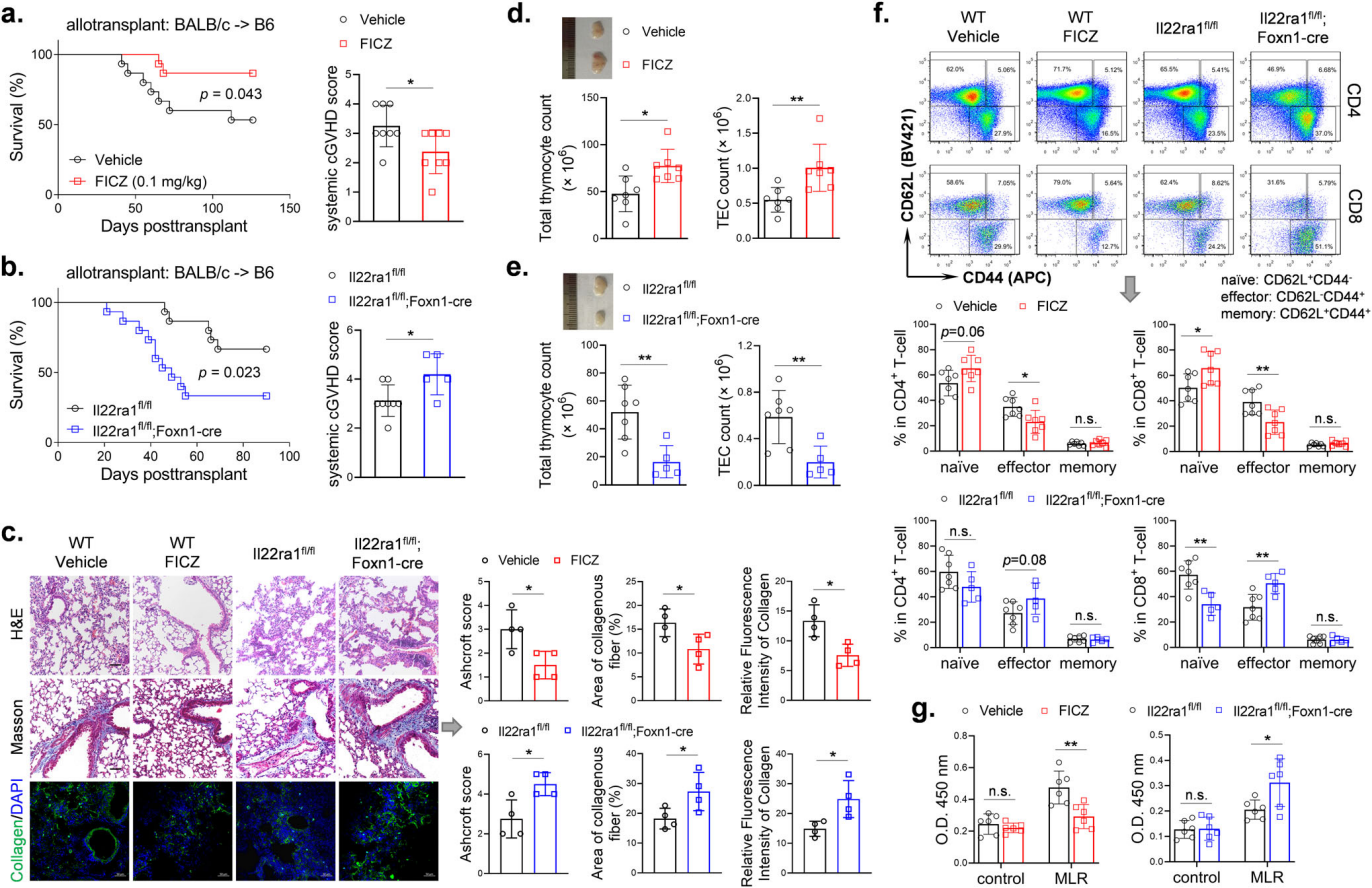
Targeting AHR or IL-22RA1 had an impact on thymus regeneration, T-cell reconstitution and pathogenesis of murine cGVHD following allo-HCT. In the cGVHD models, BALB/c mice were used as donors. Wild type C57BL/6 (**a**), B6-*Il22ra1*^*fl/fl*^ and B6-*Il22ra1*^*fl/fl*^*;Foxn1-cre* mice (**b**) were recipients. Wild type C57BL/6 recipients were treated by vehicle or FICZ thrice weekly for 2 weeks. **a**, **b** Survival (*n* = 15 in each group) and cGVHD score (Day 60) of recipient mice were monitored. **c** Histological analyses (Day 90): H&E staining, Masson staining and collagen immunofluorescence staining (*n* = 4). Ashcroft score was determined by inflammation and fibrosis. Area of collagenous and fluorescence intensity was analyzed using ImageJ. Scale bar: 50 μm. **d**, **e** At day 90, thymus cells were counted as described (*n* = 7 or 5). **f** At day 90, blood samples were collected and flow cytometry was used to analyze T-cell subpopulations (*n* = 7 or 5). **g** At day 90, T cells were isolated from spleen cells of recipients. Lymphocytes were isolated from spleen cells of normal C57BL/6 mice. The isolated recipients’ T cells were cultured alone (control) or co-cultured (MLR) with normal lymphocytes (1:1) for 72 h. Cell viability was detected by CCK-8 assay (*n* = 7 or 5). Survival is compared using log-rank test. Data are mean ± SD, compared using one-way ANOVA test or Student’s *t* test. *, *p* < 0.05; **, *p* < 0.01; n.s., not significant.

Finally, we used the BALB/c → C57BL/6 murine cGVHD model to test whether targeting thymus regeneration had an impact on the pathogenesis of cGVHD which is an autoimmune-like complication following allo-HCT. Giving FICZ increased survival and decreased cGVHD symptoms of wild-type recipients (Fig. 6a). On the contrary, Foxn1-cre-mediated IL-22RA1 knockout in recipient mice decreased survival of recipients and increased cGVHD symptoms (Fig. 6b). Lung is a primary target organ of cGVHD. Histological analyses were performed to assess lung cGVHD. FICZ reduced inflammation and alveolar thickening in the lung as indicated by H&E staining and Ashcroft score. FICZ also decreased fibrosis in lung tissues as indicated by Masson staining and immunofluorescence staining of collagen. *Il22ra1*KO increased these pathological characteristics (Fig. 6c).

We further explored whether the impacts on cGVHD were associated with thymus regeneration and T-cell immune reconstitution. FICZ increased cell counts of total thymocytes and TECs of recipients, whereas *Il22ra1*KO decreased counts of these cells (Fig. 6d, e and Supplementary Fig. 4). Neither FICZ nor *Il22ra1*KO changed T-cell counts in peripheral blood (data not shown), but these interventions had significant impacts on the proportion of T-cell subpopulations in blood. FICZ increased the percent of naïve T cells and decreased percent of effector T cells in both CD4^+^ T cells and CD8^+^ T cells. *Il22ra1*KO decreased the percent of naïve T cells and increased the percent of effector T cells in CD8^+^ T cells (Fig. 6f and Supplementary Fig. 1). To test the autoimmune potential of recipients’ T cells, we performed mixed lymphocyte reaction (MLR) by isolating recipients’ T cells and co-culturing the T cells with lymphocytes isolated from normal C57BL/6 mice. T cells from vehicle-treated and FICZ-treated wild-type recipients, without co-culturing (control), showed similar viability after 72 h culture. In the co-culturing cells, T cells from FICZ-treated wild-type recipients showed lower viability than those from vehicle-treated ones (Fig. 6g), which indicated FICZ treatment decreased autoreactivity of recipients’ T cells. By contrast, *Il22ra1*KO increased the autoreactivity of recipients’ T cells (Fig. 6g).

## Discussion

AHR exerts important functions of regulating immune responses especially in T cells. AHR induced expressions of IL-17, IL-22, IL-10 and Aiolos in T cells which determine differentiation and effect of helper T cells and regulatory T cells^11^. These are focused on the effects in peripheral or terminally differentiated T cells. By contrast, we showed an indirect effect of TECs-expressed AHR in promoting T-cell reconstitution after thymus damage. Our findings provide a new insight how AHR regulates T-cell homeostasis from the central immune organ. Both AHR and IL-22RA1 in TECs were important for thymus regeneration after irradiation-induced acute damage. AHR regulated IL-22RA1 expression which was fundamental for IL-22-mediated thymus regeneration.

Our study showed a new mechanism regulating thymus regeneration. AHR exerted multiple functions in T cells from other studies^15^. We found TECs-expressed AHR was important for recovery of thymus cells after damage, through a mechanism that AHR transcriptionally regulated IL-22RA1 gene expression in TECs. Others reported AHR-regulated IL-10 receptor expression on intestinal epithelial cell which was important for maintaining homeostasis of intestinal epithelia^16^. Knockout or pharmacological inhibition of AHR reduced IL-22RA1 mRNA and protein levels in TECs which indicated AHR was crucial for IL-22RA1 gene expression. However, overexpression or agonist of AHR did not significantly upregulate the transcription activity of IL-22RA1 gene in mTEC1 cells. Others reported STAT3 was important for maintaining IL-22RA1 expression^14^. We proved binding of AHR and STAT3 in vitro and they bound to the same region of IL-22RA1 promotor. Simultaneous activation of the two transcription factors increased transcription activity and mRNA level of IL-22RA1 gene. The STAT3 inhibitor stattic attenuated the effect of FICZ in promoting thymus regeneration after irradiation. These results indicated AHR probably cooperated with STAT3 to increase IL-22RA1 expression. AHR also shows similar regulatory functions in immune cells. AHR cooperates with RORγt to drive IL-22 expression in Th17 cells^11^. AHR was reported to induce IL-10 and IL-21 expression in cooperation with c-Maf in type 1 regulatory T cells^11^. FICZ alone increased IL-22RA1 mRNA level to 1.3 – 1.5 times in mTEC1 cells, whereas the number was 4.5 times in the thymus of irradiated mice. The efficacy of FICZ seems to be higher in increasing IL-22RA1 mRNA level in vivo. The possible reason was irradiation also induced activation of STAT3 in TECs which could cooperates with FICZ, however, FICZ alone in mTEC1 cell culture did not increase activation of STAT3. In addition, STAT3 is responsible for IL-22RA1 intracellular signaling transduction^10^, which shows a possible positive feedback loop of IL-22RA1 and STAT3 interaction. Another possible reason is the AHR variants in different mouse strains. There are four phenotypic alleles of murine Ahr gene^17^. Ahr^b1^ (for C57BL/6) and Ahr^b2^ (for BALB/c) have similar ligand-binding affinity, but the two types show distinct single nucleotide polymorphisms which might contribute to the discrepancy between the in vivo and in vitro experiments. The mTEC1 cell line is of BALB/c origin, whereas we used C57BL/6 mice in animal models due to the availability of C57BL/6-derived genetically modified mice.

Dysfunction of the thymus can result in the release of autoreactive T cells and cause autoimmune diseases^7,18^. cGVHD, a later-phase complication of allo-HCT, is characterized by autoimmunity and organ fibrosis which severely worsen the life quality of allotransplant patients^13^. There are several evidences showing the correlation between defective thymus function and the occurrence of cGVHD^19,20^. TECs are important for proliferation and acquiring self-tolerance of developing thymocytes^6^. Improving TECs recovery possesses a rationale for better reconstitution of T cells. We showed targeting AHR with FICZ improved thymus regeneration and decreased the severity of murine cGVHD. TECs-specific knockout of IL-22RA1 resulted in poor thymus regeneration and increased cGVHD as evidenced by pathological features in lung tissues. We showed the impacts on cGVHD were associated with T-cell immune reconstitution. Naïve T cells are resting, whereas effector T cells are activating and considered dominant mediators of GVHD^13,21^. FICZ treatment increased naïve T cells but decreased effector T cells. TECs-specific knockout of IL-22RA1 showed the quite contrary effects. The MLR assay directly proved the autoimmune potential of recipients’ T cells, which were significantly altered by targeting AHR or IL-22RA1. AHR also shows effects in other autoimmune diseases^15^. AHR activation by a non-toxic agonist increased the immunoregulatory function of Treg cells and ameliorated inflammatory bowel disease^22^. Gut commensal bacteria-derived tryptophan metabolites can also activate AHR and contribute to the homeostasis of gut epithelia in inflammatory bowel diseases^23^. It was reported that an endogenous AHR ligand reduced experimental autoimmune encephalomyelitis by effects on dendritic cells and T cells^24^.

IL-22 was proved with protective effects in allo-HCT. IL-22 protected intestinal epithelial cells and decreased gut GVHD^25^. The clinical trial using recombinant human IL-22 to treat gastrointestinal GVHD reported the results recently, which showed IL-22 in combination with systemic corticosteroids alleviated GVHD-associated gut dysbiosis^26^. We and others previously reported IL-22 exerted an effect in improving thymus regeneration following allo-HCT^9,27^. Thus, using IL-22 might be a promising strategy for controlling GVHD in allo-HCT. It would be important to increase the efficacy of IL-22, and AHR is a possible combinatory target here. AHR induced IL-22RA1 expression which warranted the effect of IL-22.

There are several AHR ligands with different potential in activating AHR pathway and in regulating immune cell functions. Tetrachlorodibenzo-p-dioxin (TCDD), ITE and FICZ increased regulatory T cells and decreased effector T cells in a murine alloresponse model. These ligands all showed direct impact on T-cell differentiation with ITE as the most effective one, however, low dose FICZ did not show such significant effects in the alloresponse^28^. Low-dose FICZ is used in our study to avoid the effect in directly regulating T-cell differentiation which is a confound factor in interpreting the effect of FICZ in thymus regeneration. TCDD shows a sustained effect in AHR activation for up to 2 weeks, in contrast, FICZ is metabolized rapidly in vivo. Thymus regeneration is most vigorous during the first 1-2 weeks following irradiation^9^. Using the short-acting ligand is easier to timely control thymus regeneration and might avoid other side effects caused by long-term action. ITE acts not only on T cells, but also directly on dendritic cells to induce tolerogenic dendritic cells that support FoxP3-positive Treg differentiation^24^. Dietary AhR ligand indole-3-carbinol reduced Th17 cells while promoting Treg, and attenuated delayed-type hypersensitivity response to methylated BSA. In contrast, FICZ exacerbated the delayed-type hypersensitivity response and promoted Th17 cells^29^. TCDD and FICZ increased the expression of IL-10 and IL-6 in LPS-treated macrophages, whereas indole-3-carbinol decreased these cytokines^30^. Regarding the impact of AHR ligands on thymus, TCDD was reported to induce thymus involution in physiological conditions, and thymocytes are responsible for TCDD-induced thymic atrophy^31^. Irradiation-induced acute injury triggered prompt regenerative process of the thymus, in which TECs-expressing AHR shows a beneficial impact. In addition, low-dose FICZ was not reported to induce thymus involution in the previous studies. Our study showed low dose FICZ effectively promoted thymus regeneration after irradiation-induced acute injury.

In addition to TECs, thymus regeneration can be regulated by other types of intrathymic cells such as keratinocytes and dendritic cells. AHR in these Foxn1-expressing cells might also contribute to thymus regeneration. Activation of AHR in thymic dendritic cells is responsible for TCDD-induced alterations in thymus^32^. The possible Foxn1-expression in intrathymic dendritic cell might contribute to AHR-mediated thymus regeneration in our study, however, this possible Foxn1-expression has not been proved so far. Previous studies show that Foxn1 is expressed in TECs, some keratinocytes and hair follicle cells^33,34^.

Our study has limitations. STAT3 is a possible cooperative partner of AHR in thymus regeneration. However, STAT3 is considered pro-inflammatory in GVHD because it mediates signal transduction of several pro-inflammatory cytokines such as IL-6 and IL-21^35^, and activation of STAT3 has unfavorable impacts on GVHD^36,37^. There is a possible competition between FICZ and STAT3 inhibitor regarding the control of cGVHD. STAT3 inhibitor decreases cGVHD while exerting the potential of blocking thymus regeneration. On the contrary, activation of STAT3 might facilitate thymus regeneration but with the risk of increasing cGVHD. Perhaps future study would shed new light on the combined use of STAT3 inhibitor and FICZ through investigating the administration time point and dose of STAT3 inhibitor which preferentially reduces actions of pro-inflammatory cytokines without significant blockade on thymus regeneration and vice versa. Other assays such as proteomics might identify new cooperative factors of AHR which would have a favorable impact added on AHR. Although our cGVHD mice showed significant fibrosis, it was not enough to represent other aspects of autoimmune diseases such as autoantibody secretion and B-cell activation.

In conclusion, we reported an unrecognized function of AHR in thymus regeneration. TECs-expressed AHR transcriptionally regulated IL-22RA1 expression on TECs and mediated thymus regeneration after damage. Giving the AHR agonist FICZ accelerated thymus regeneration and decreased cGVHD in murine allo-HCT.

## Methods

### Mice

Wild type C57BL/6 and BALB/c mice (6–8 w, 18–20 g) were purchased from Charles River Laboratories (Vital River, Beijing, China). B6-*Ahr*^*fl/fl*^*;Foxn1-cre* mice and B6-*Il22ra1*^*fl/fl*^*;Foxn1-cre* mice were ordered from Cyagen (Suzhou, China). Procedures regarding animal care and experiments were approved by Medical Ethics Committee of Xuzhou Medical University. Mice were anesthetized by isoflurane before injection and blood sample collection. To collect tissue samples, mice were anesthetized by isoflurane and euthanized. Blood samples were collected from mice orbital vein with EDTA as anticoagulant.

### Cells

The murine thymic epithelial cell line mTEC1 (derived from BALB/c mice) was kindly provided by Prof. Yu Zhang (Peking University, Beijing). mTEC1 cells were cultured in DMEM medium (Thermo Fisher Scientific, Waltham, MA, USA) containing 10% FBS. T cells were isolated from spleen cells using the EasySep Mouse T Cell Isolation Kit (19851, STEMCELL Technologies, Shanghai, China). Lymphocytes were isolated from spleen cells using the Lymphocyte Separation Medium (7211011, DAKEWE, Shenzhen, China). The isolated T cells were cultured alone or co-cultured with lymphocytes (1:1) in RPMI-1640 medium (Sigma-Aldrich, Shanghai, China) containing 10% FBS for 72 h.

### Reagents

FICZ (HY-12451), CH-223191 (HY-12684), stattic (HY-13818) and colivelin (HY-P1061A) were purchased from MedChemExpress (Monmouth Junction, NJ, USA). Recombinant murine IL-22 (rmIL-22) was from PeproTech (AF-210-22, Rocky Hill, NJ, USA). Dose and concentration of FICZ were determined according to others’ reports^16,38^. For in vivo experiments, vehicle was 20% PEG water solution which was used to dilute reagents. For in vitro experiments, vehicle was PBS buffer containing the same percent of DMSO which was used to dilute reagents.

### Acute thymus injury model

Acute thymus injury was induced as described previously^39^. Mice were treated by 5.5 Gy total body irradiation (TBI) with ^137^Cs source. Following irradiation, mice were intraperitoneally injected with vehicle, FICZ, CH-223191, stattic or rmIL-22 thrice weekly for 2 weeks or without any treatment.

### Thymus cell preparation

To disperse thymus cells, freshly dissected thymii were cut into small pieces and treated by digestion with 0.5 mg/ml collagenase D, 1 mg/ml DNase I and 1 mg/ml Dispase. Enzymes were from Sigma-Aldrich (Shanghai, China). The dispersed cells were re-suspended in RPMI-1640 medium containing 2% FBS and 5 mM EDTA. To isolate thymic stromal cells, thymus was mildly ground and shaken to release thymocytes, and the remained thymus gland containing connective tissue was collected.

### Flow cytometry

To analyze subpopulations of thymus cells, the obtained single-cell suspension was stained with anti-CD45 (103132, BioLegend, San Diego, CA, USA), anti-CD4 (100510, BioLegend), anti-CD8a (100708, BioLegend) and anti-EpCAM (563478, BD Biosciences, San Jose, CA, USA). The following markers were used to define different subpopulations. TECs: CD45^-^EpCAM^+^; double positive (DP) thymocytes: CD45^+^CD4^+^CD8^+^; single positive (SP) thymocytes: CD45^+^CD4^+^CD8^-^ and CD45^+^CD4^-^CD8^+^; double negative (DN) thymocytes: CD45^+^CD4^-^CD8^-^. To analyze T-cell subpopulations in blood, cells were staining with anti-CD3e (100328, BioLegend), anti-CD4 (RM4-5), anti-CD8a (53-6.7), anti-CD44 (17-0441-83, Thermo Fisher Scientific) and anti-CD62L (104435, BioLegend). CD4^+^ T cells and CD8^+^ T cells were gated on CD3^+^CD4^+^CD8^-^ and CD3^+^CD8^+^CD4^-^ subpopulations, and were further analyzed for expressions of CD44 and CD62L. Naïve T cells: CD62L^+^CD44^-^; effector T cells: CD62L^-^CD44^+^; memory T cells: CD62L^+^CD44^+^. Antibodies were used at a working concentration of 1 μg/ml. Cells were acquired on an LSRFortessa flow cytometer (BD Biosciences), and analyzed using FlowJo 7.6 software.

### Quantitative PCR

RNA was extracted from tissues or cells using Trizol reagent (Thermo Fisher Scientific). cDNA synthesis was performed using PrimeScript™ RT reagent Kit (RR037A, TAKARA, Kusatsu, Japan) as described^40^. Quantitative PCR was performed with the LightCycler 480 SYBR Green I Master kit (4887352001, Roche, Mannheim, Germany). The following primers were used: IL-22 (TCGCCTTGATCTCTCCACTC and GCTCAGCTCCTGTCACATCA), IL-22RA1 (CAGCGGATCACCCAGAAGTT and GCGGTTTGATGGTAGTGTGC), Ahr (CACAGAGTTAGACCGCCTGG and GCCATTCAGCGCCATCAAAG), CYP1A1 (TAACCATGACCGGGAACTGT and TTCGCTTGCCCAAACCAAAG), GAPDH (TTGATGGCAACAATCTCCAC and CGTCCCGTAGACAAAATGGT). GAPDH was the normalization gene. Relative mRNA levels were –ΔΔC_T_ values.

### Western blot

Proteins were extracted from cells or tissues using Cell Lysis Buffer (9803, Cell Signaling Technology, Danvers, MA, USA). The following antibodies were used. STAT3 (4904) and p-STAT3 (9145) were from Cell Signaling Technology. IL-22RA1 (13462-1-AP), AHR (67785-1-Ig) and β-actin (20536-1-AP) were from Proteintech (Carlsbad, CA, USA). β-actin antibody was used at the dilution of 1:2000, and the other antibodies were used at 1:1000. All blots were processed in parallel and derive from the same experiments. Uncropped scans are in the Supplementary Figures.

### Immunofluorescence

Immunofluorescence was performed on cryosections of thymus and lung. To detect IL-22RA1, slides were incubated with the first antibody rabbit-anti-IL-22RA1 (13462-1-AP, Proteintech, dilution at 1:100) overnight at 4 °C. CoraLite488-conjugated Goat Anti-Rabbit IgG (SA00013-2, Proteintech, dilution at 1:200) was used as the second antibody for visualization. To detect collagen, rabbit-anti-Collagen (14695-1-AP, Proteintech, dilution at 1:100) and CoraLite488-conjugated Goat Anti-Rabbit IgG were used. DAPI was used to stain nuclei. Images were acquired and analyzed on a Zeiss 880 confocal microscope.

### Knockdown of AHR gene

Mouse AHR-targeted short hairpin RNAs (Ahr shRNA) was designed referring to mouse AHR mRNA sequence (NM_013464). Three target sites were designed. Target 1: GCTCAGGAATTTCCCTACAAA; Target 2: CATCGACATAACGGACGAAAT; Target 3: AGAGCTCTTTCCGGATAATAA. An unrelated sequence TTCTCCGAACGTGTCACGT was used as negative control. The lentiviral vector GV493 (Genechem, Shanghai, China) was used to express these shRNAs and the lentivirus was packaged consequently (Genechem). The mTEC1 cells were stably transduced with lentivirus by selection with puromycin. Our preliminary experiments showed Target 3 most significantly decreased AHR mRNA level which was around 30% of negative control.

### Overexpression of AHR gene

The CDS sequence of mouse AHR mRNA (NM_013464) was synthesized and subcloned to lentiviral vector GV358 (Genechem). Lentivirus was packaged and stable transduction in mTEC1 cells was performed as described above.

### Chromatin immunoprecipitation (ChIP)

The Enzymatic ChIP Kit (9003, Cell Signaling Technology) was used to perform ChIP analysis. Chromatin fragmentation was performed per manufacturer’s instructions. Fragmented chromatin was immunoprecipitated with anti-AHR (28727-1-AP, Proteintech, dilution at 1:100) or anti-STAT3 (4904, Cell Signaling Technology, dilution at 1:100). Rabbit IgG (30000-0-AP, Proteintech or 2729, Cell Signaling Technology, dilution at 1:100) was used as a negative control. The precipitated DNA was analyzed by qPCR. The primers were designed referring to the promoter region (-1800bp — -1500bp of TSS) of IL-22RA1: GGTAATAGCAACACCACCAGT and GCTGAGATAACCACCGAAGC. Enrichment of the target DNA fragment was normalized to the content in input using C_T_ values and expressed as signal relative to input.

### Luciferase reporter assay

To construct the firefly luciferase-expressing plasmid, the promoter region (-2000bp — -1300bp of TSS) of mouse IL-22RA1 gene was synthesized and subcloned to the pGL4.10 vector (General Biol, Hefei, China). The renilla luciferase-expressing plasmid pRL-TK vector (Promega, Madison, WI, USA) was used as a normalization control. The two vectors were simultaneously transfected into mTEC1 cells using Lipofectamine 2000 (Thermo Fisher Scientific). After 12 h incubation, cells were harvested and processed with the Dual-Luciferase Reporter Assay System (E1910, Promega). Luciferase activity was detected on a Promega GloMax 96 reader.

### Immunoprecipitation

To detect binding of AHR and STAT3, total cell proteins were incubated with anti-STAT3 (4904, Cell Signaling Technology, dilution at 1:100) and anti-AHR (28727-1-AP, Proteintech, dilution at 1:100), respectively. Pull-down assay was with Protein A Magnetic Bead (LSKMAGA02, Merck, Shanghai, China). The precipitate was detected by western blot with anti-AHR (67785-1-Ig, Proteintech) and anti-STAT3 (4904, Cell Signaling Technology). Rabbit IgG (30000-0-AP, Proteintech, dilution at 1:100) was used as a negative control.

### Cell viability

To measure cell viability, cells were incubated with Cell Counting Kit-8 reagent (CK04, DOJINDO, Tokyo, Japan) for 2 h and optical density was read at 450 nm.

### Transplant

The cGVHD model was established as described^41^. BALB/c mice were used as donors. Wild type C57BL/6, B6-*Il22ra1*^*fl/fl*^ and B6-*Il22ra1*^*fl/fl*^*;Foxn1-cre* mice were recipients. The recipient mice were treated with 9.0 Gy TBI and received an injection of 5 × 10^6^ bone marrow cells and 1 × 10^6^ spleen T cells from BALB/c donors. Wild-type recipients were intraperitoneally injected with vehicle or FICZ thrice weekly for 2 weeks. The other recipients were without any treatment. Survival was monitored continuously. Systemic cGVHD symptoms were assessed according to a scoring criterion as described^41^.

### Histology

H&E staining was performed on slides of thymus and lung tissues. Masson staining (HT15, Sigma-Aldrich) was performed on lung tissue slides to assess fibrosis. Lung cGVHD was assessed by analyzing inflammation, alveolar thickening and fibrosis with Ashcroft score^42^.

### Statistics

Data are mean ± standard deviation. Individual values are displayed in the figures. Survival is compared using log-rank test. Number (n) represents independent values from distinct samples. Comparisons of means are performed using two-tailed unpaired Student’s *t* test or one-way ANOVA test. *P* values < 0.05 are considered statistically significant.

### Reporting summary

Further information on research design is available in the Nature Research Reporting Summary linked to this article.

### Supplementary information

Supplementary Figures 1-10

Reporting Summary

## Data Availability

Data of this study are available from the corresponding author on reasonable request.
