# Supplementary material for: Aryl hydrocarbon receptor regulates IL-22 receptor expression on thymic epithelial cell and accelerates thymus regeneration
Source: NPJ Regen Med. 2023 Nov 8;8:64. doi: 10.1038/s41536-023-00339-7 (PMC10632505; doi:10.1038/s41536-023-00339-7)
Supplement: Supplementary file 1 — Supplementary Figures 1-10 [file 41536_2023_339_MOESM1_ESM.pdf]

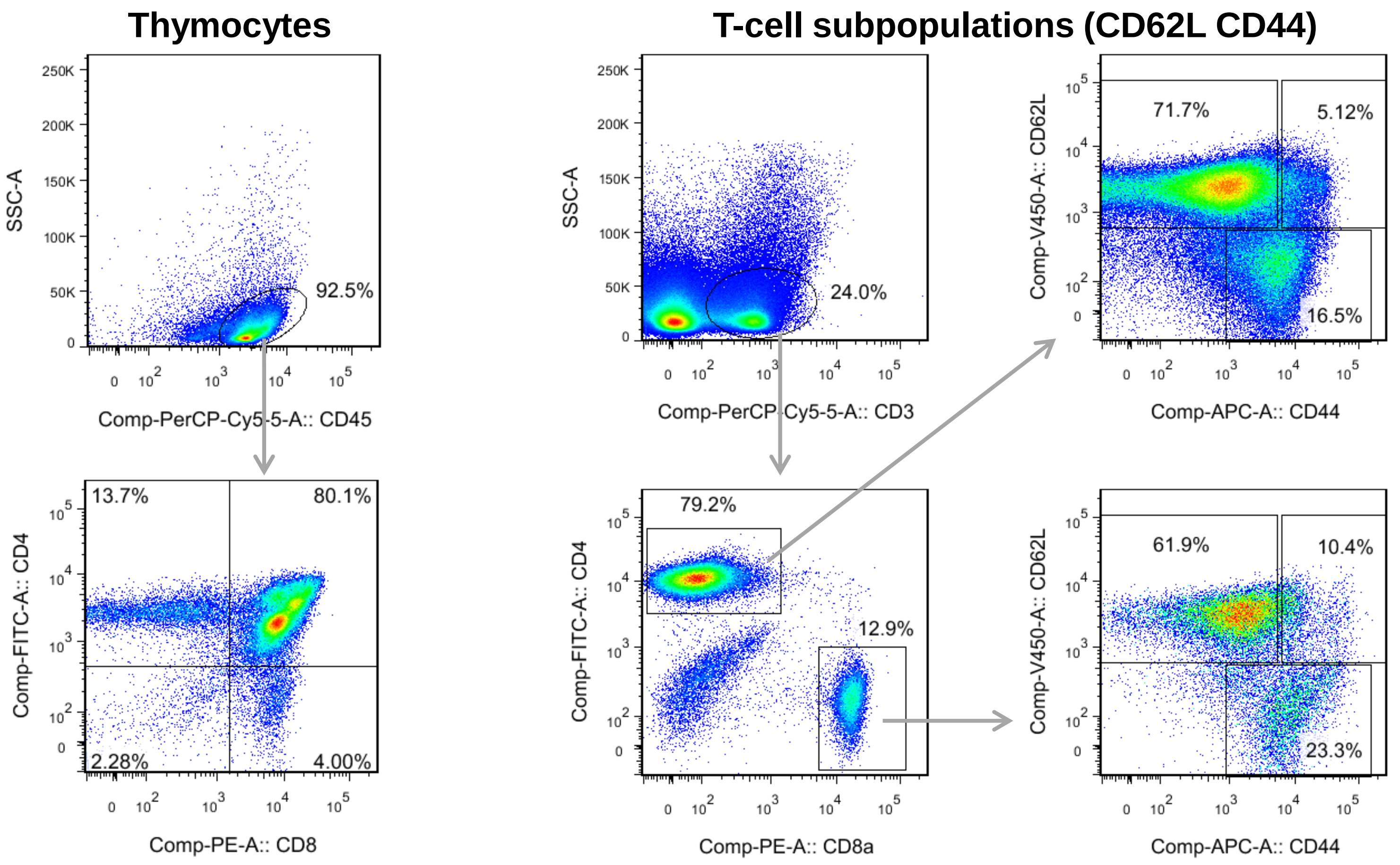

Supplementary Figure 1. Gating strategy in flow cytometric analysis.

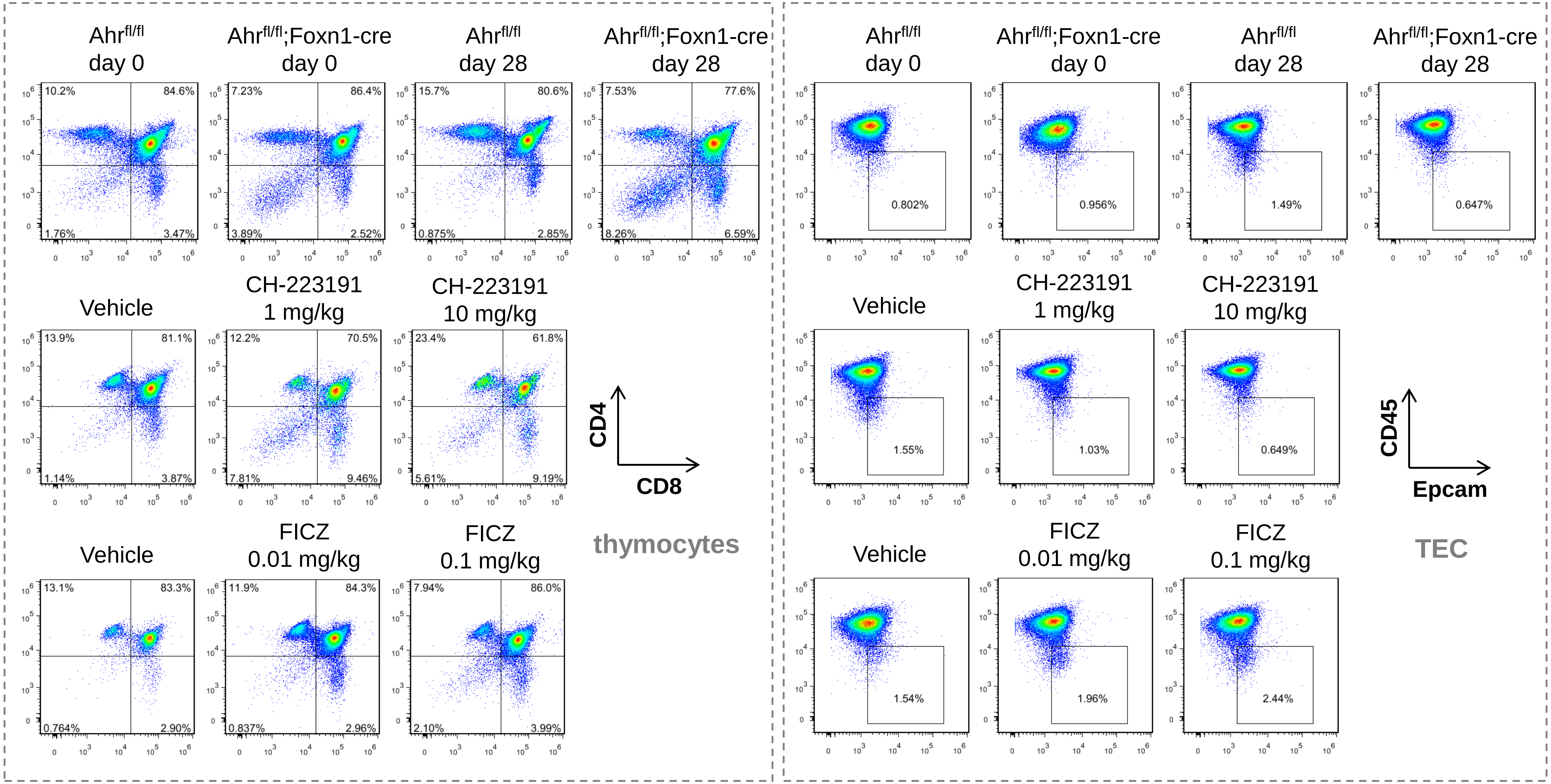

Supplementary Figure 2. Flow cytometric analysis of thymus cells isolated from wild type C57BL/6 mice, B6-*Ahr*$^{fl/fl}$ mice and B6-*Ahr*$^{fl/fl}$*;Foxn1-cre* mice as described in Fig. 1. CD4 and CD8 subpopulations were gated on CD45^{+} cells. Epcam and CD45 subpopulations were total thymus cells.

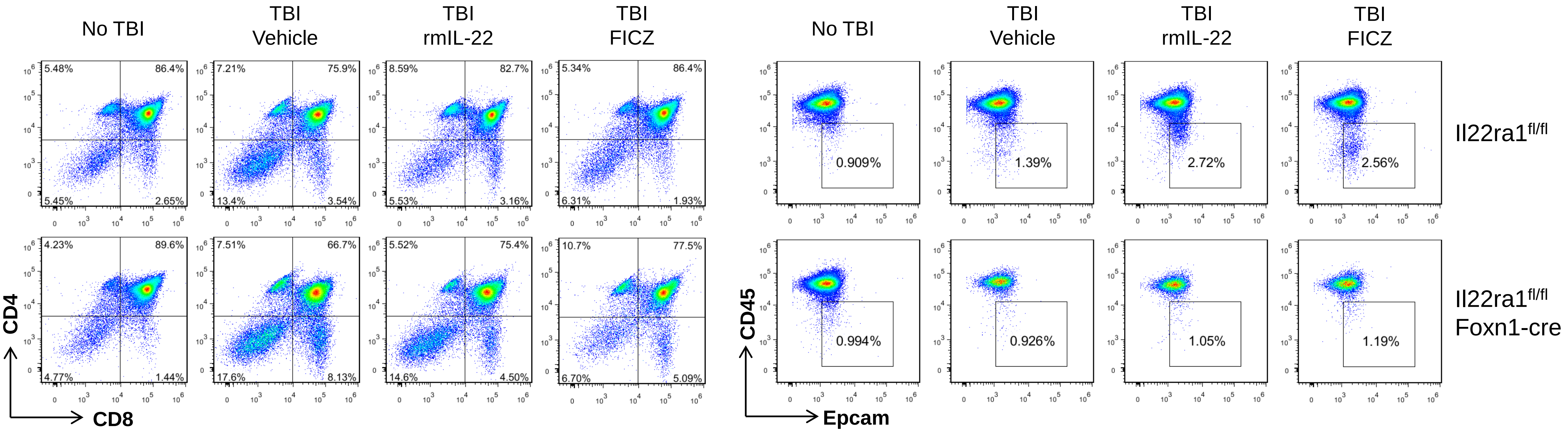

Supplementary Figure 3. Flow cytometric analysis of thymus cells isolated from B6-*Il22ra1*$^{fl/fl}$ mice and B6-*Il22ra1*$^{fl/fl}$;*Foxn1-cre* mice as described in Fig. 5c.

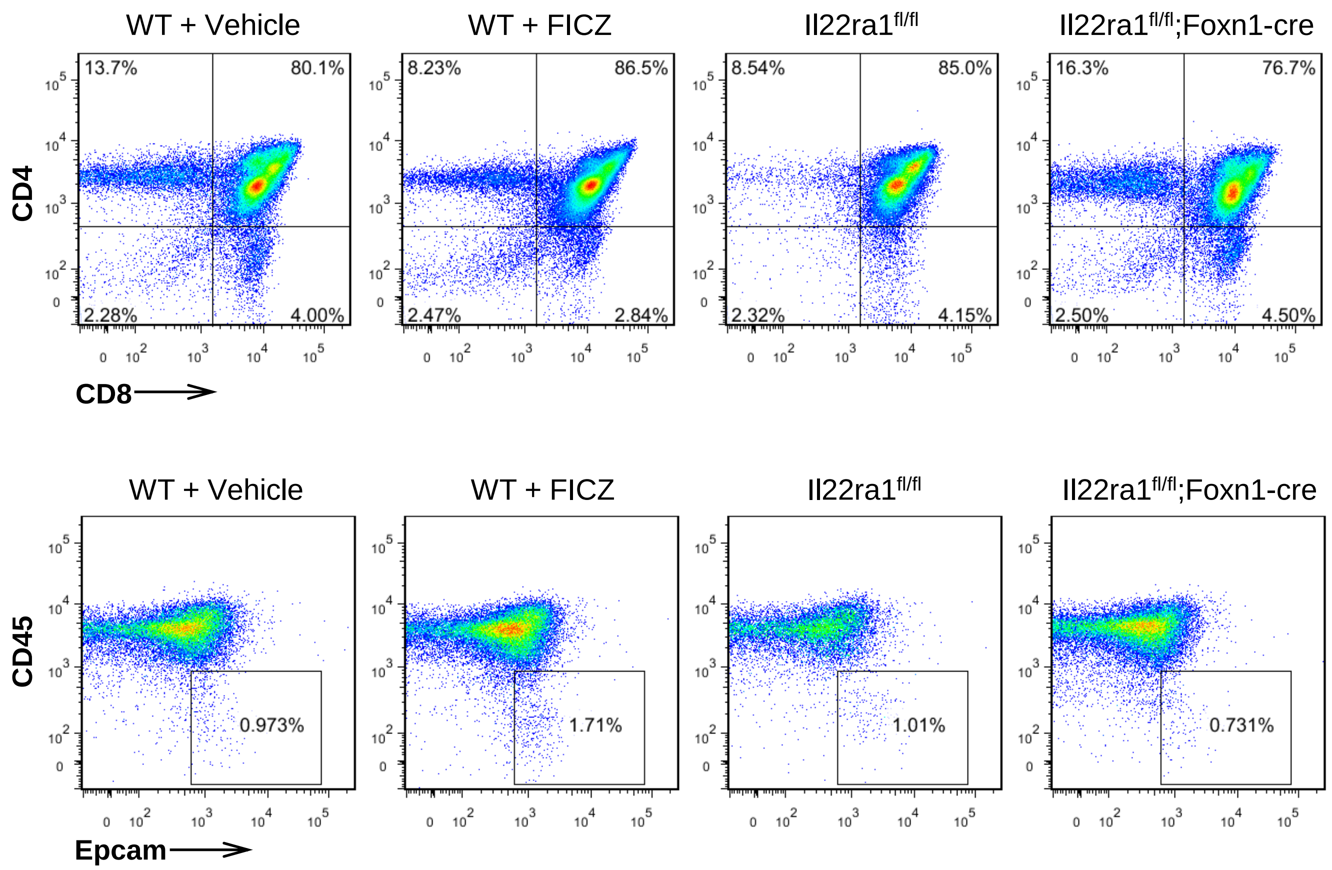

Supplementary Figure 4. Flow cytometric analysis of thymus cells isolated from recipient mice as described in Fig. 6d and 6e.

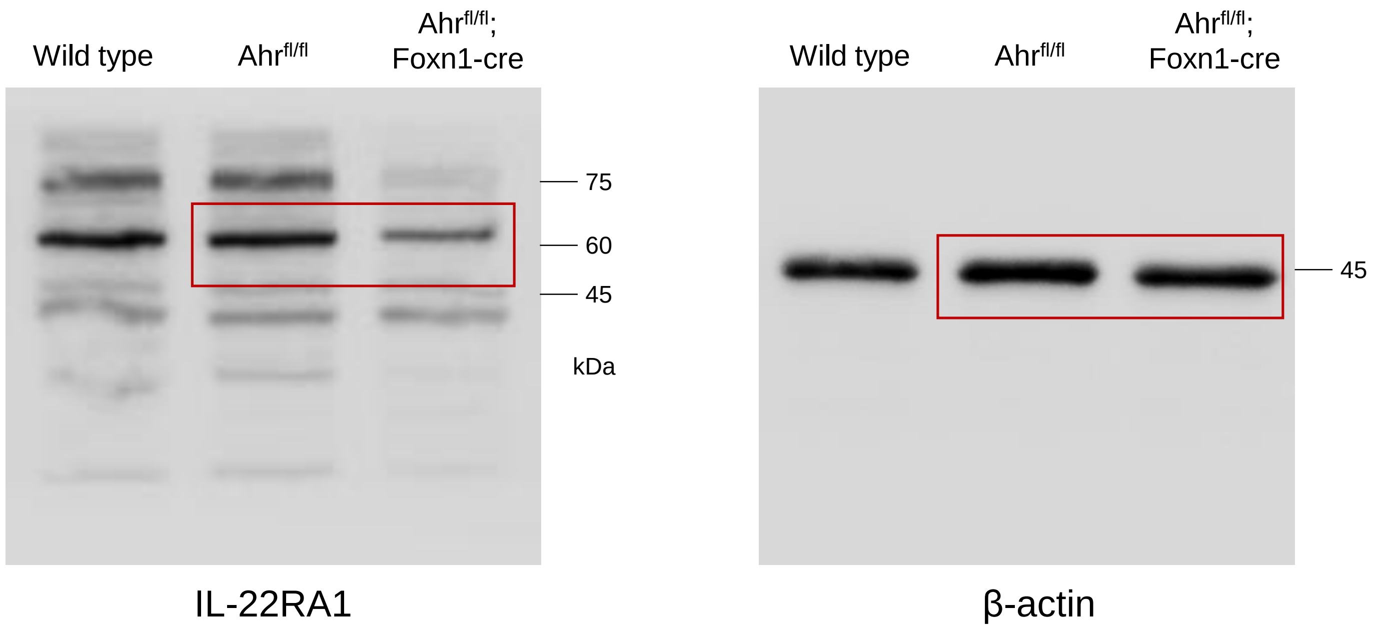

Supplementary Figure 5. Uncropped scans of Fig. 2d.

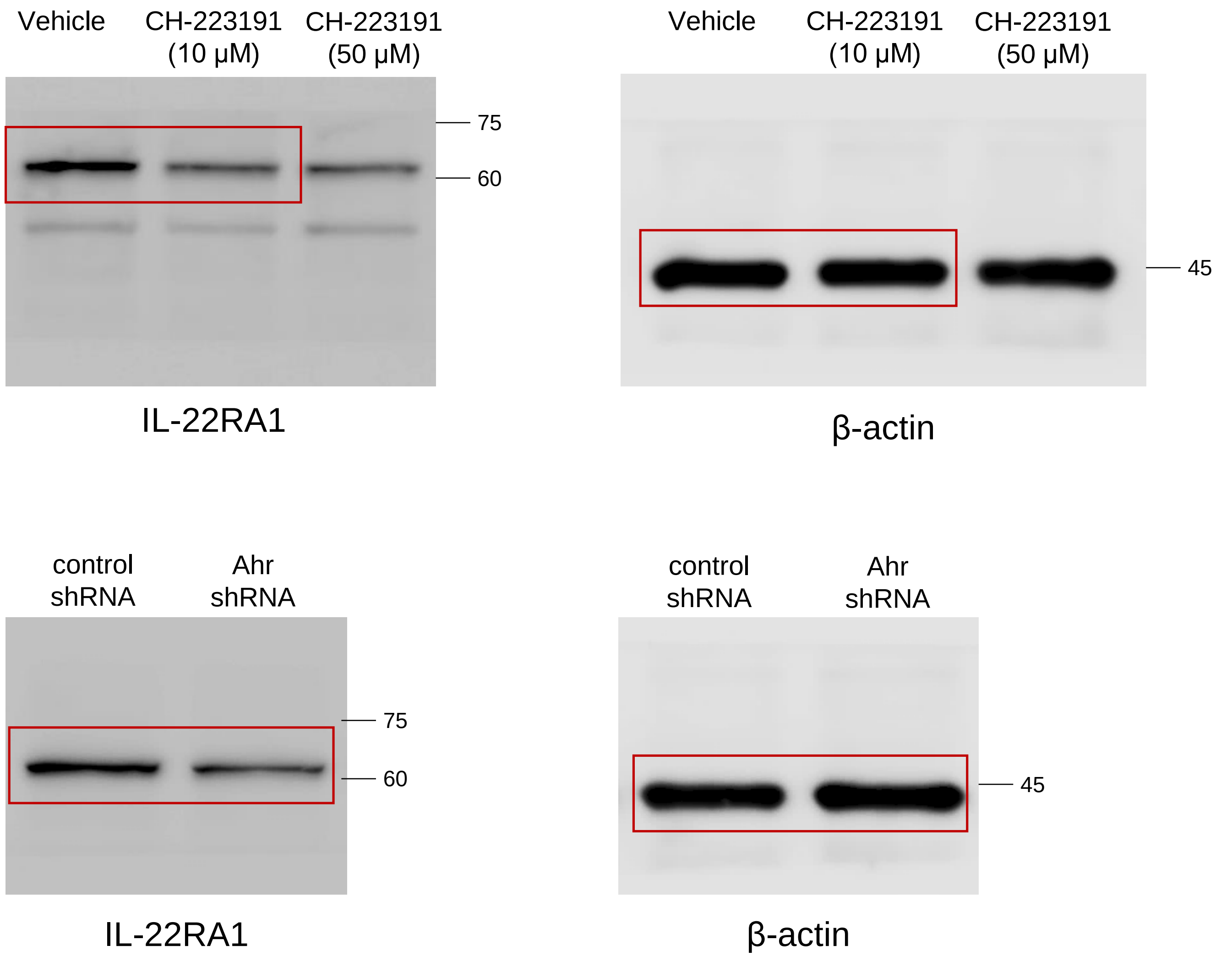

Supplementary Figure 6. Uncropped scans of Fig. 3d.

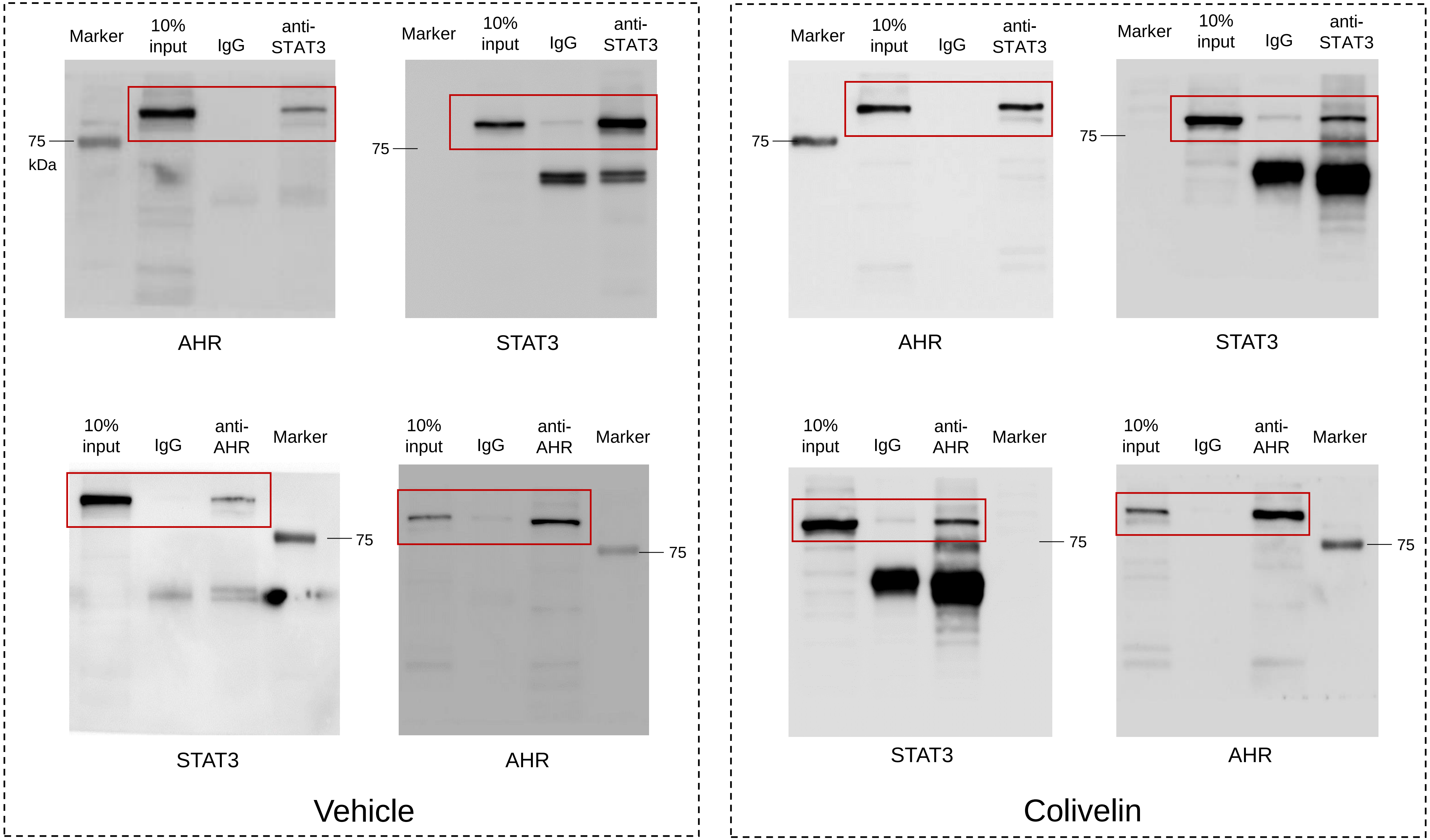

Supplementary Figure 7. Uncropped scans of Fig. 4a.

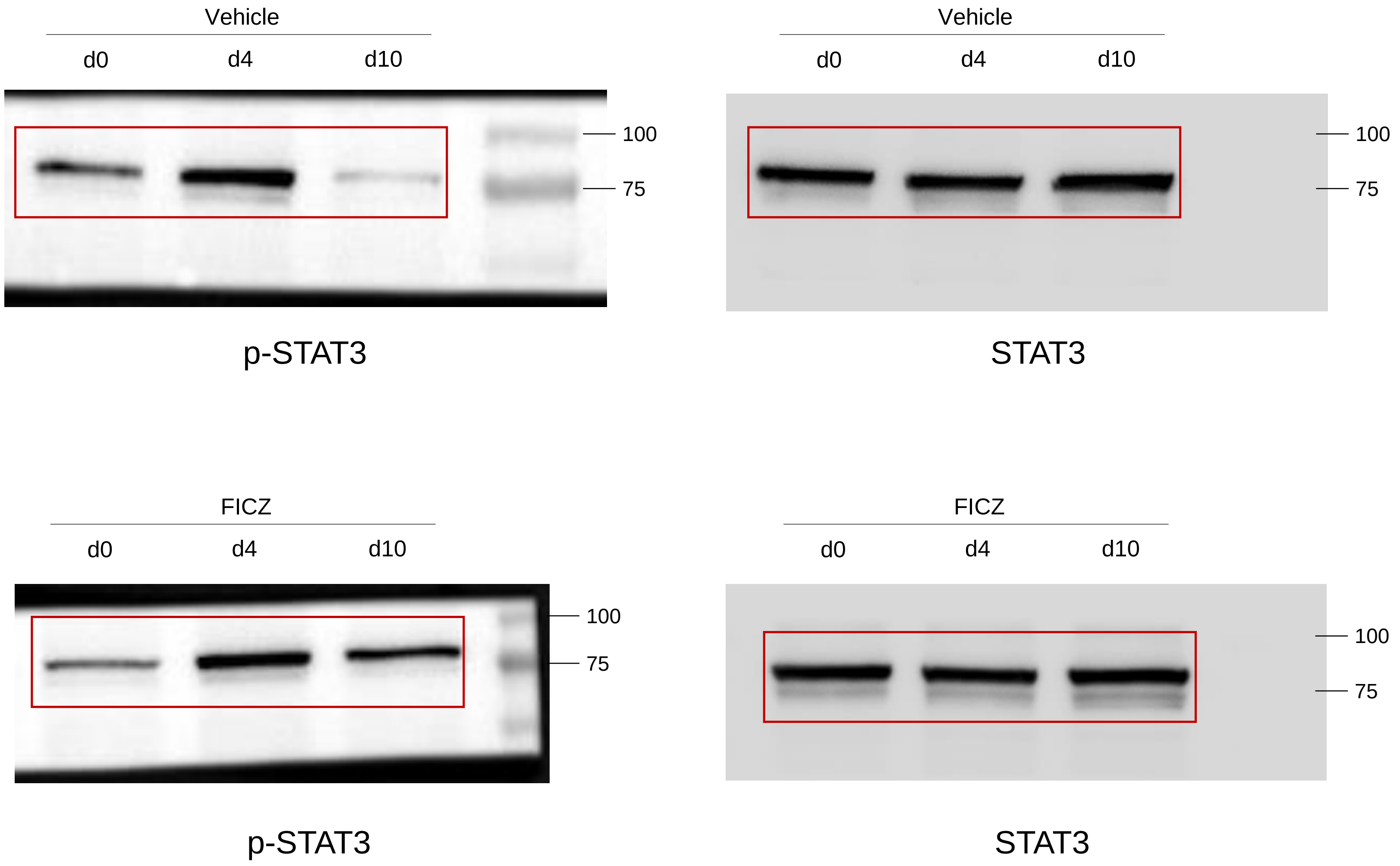

Supplementary Figure 8. Uncropped scans of Fig. 4f.

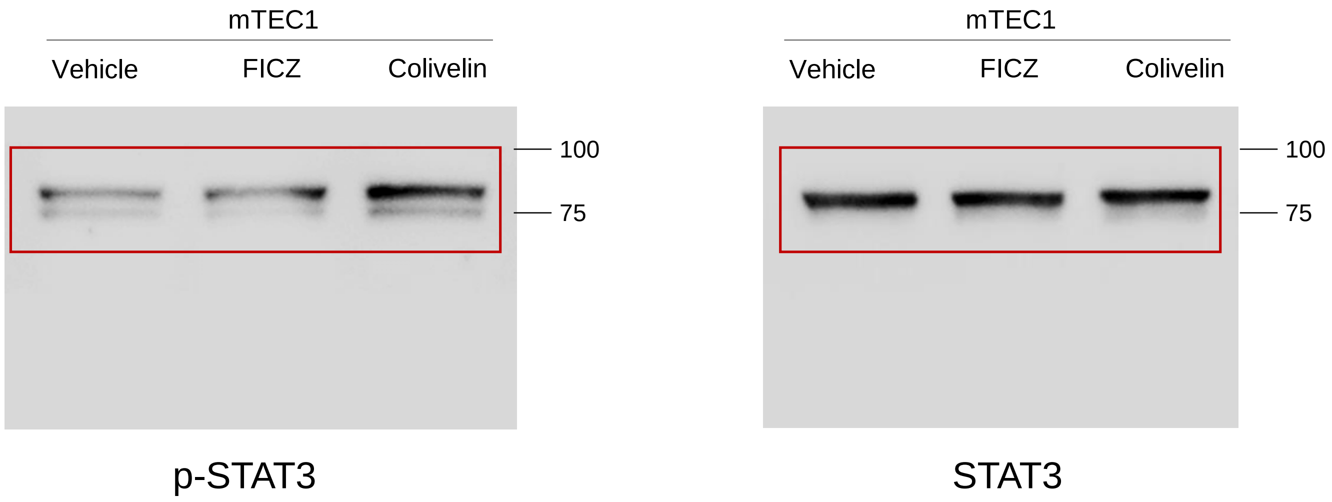

Supplementary Figure 9. Uncropped scans of Fig. 4g.

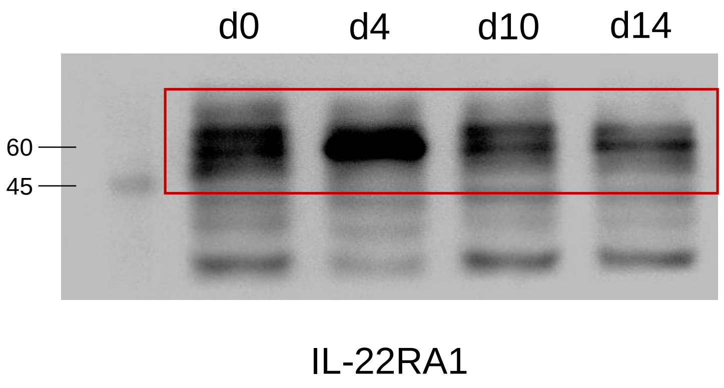

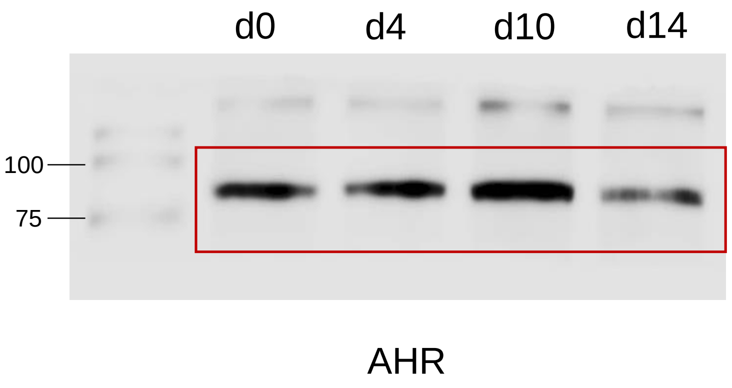

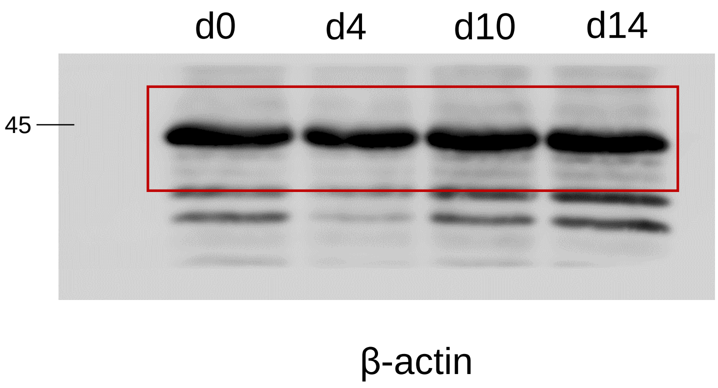

Supplementary Figure 10. Uncropped scans of Fig. 5b.
