# Supplementary material for: Aryl hydrocarbon receptor regulates IL-22 receptor expression on thymic epithelial cell and accelerates thymus regeneration
Source: NPJ Regen Med. 2023 Nov 8;8:64. doi: 10.1038/s41536-023-00339-7 (PMC10632505; doi:10.1038/s41536-023-00339-7)
Supplement: Supplementary file 2 — Reporting Summary [file 41536_2023_339_MOESM2_ESM.pdf]

nature portfolio

Corresponding author(s): Bin Pan, Kailin Xu

Last updated by author(s): Sep 7, 2023

Reporting Summary

Nature Portfolio wishes to improve the reproducibility of the work that we publish. This form provides structure for consistency and transparency in reporting. For further information on Nature Portfolio policies, see our Editorial Policies and the Editorial Policy Checklist.

Statistics

For all statistical analyses, confirm that the following items are present in the figure legend, table legend, main text, or Methods section.

n/a	Confirmed	
☐	☒	The exact sample size (*n*) for each experimental group/condition, given as a discrete number and unit of measurement
☐	☒	A statement on whether measurements were taken from distinct samples or whether the same sample was measured repeatedly
☐	☒	The statistical test(s) used AND whether they are one- or two-sided<br>*Only common tests should be described solely by name; describe more complex techniques in the Methods section.*
☒	☐	A description of all covariates tested
☒	☐	A description of any assumptions or corrections, such as tests of normality and adjustment for multiple comparisons
☐	☒	A full description of the statistical parameters including central tendency (e.g. means) or other basic estimates (e.g. regression coefficient) AND variation (e.g. standard deviation) or associated estimates of uncertainty (e.g. confidence intervals)
☐	☒	For null hypothesis testing, the test statistic (e.g. *F*, *t*, *r*) with confidence intervals, effect sizes, degrees of freedom and *P* value noted<br>*Give P values as exact values whenever suitable.*
☒	☐	For Bayesian analysis, information on the choice of priors and Markov chain Monte Carlo settings
☒	☐	For hierarchical and complex designs, identification of the appropriate level for tests and full reporting of outcomes
☒	☐	Estimates of effect sizes (e.g. Cohen's *d*, Pearson's *r*), indicating how they were calculated

Our web collection on statistics for biologists contains articles on many of the points above.

Software and code

Policy information about availability of computer code

Data collection	BD FACSDiva v8.0.1, LightCycler@480 SW1.5, ZEN 2.3 (black edition), GloMax@96
Data analysis	FlowJo 7.6, LightCycler@480 SW1.5, ImageJ, ZEN 2.6 (blue edition), GraphPad 6.0

For manuscripts utilizing custom algorithms or software that are central to the research but not yet described in published literature, software must be made available to editors and reviewers. We strongly encourage code deposition in a community repository (e.g. GitHub). See the Nature Portfolio guidelines for submitting code & software for further information.

Data

Policy information about availability of data

All manuscripts must include a data availability statement. This statement should provide the following information, where applicable:

- Accession codes, unique identifiers, or web links for publicly available datasets
- A description of any restrictions on data availability
- For clinical datasets or third party data, please ensure that the statement adheres to our policy

Data of this study are available from the corresponding author on reasonable request.

Research involving human participants, their data, or biological material

Policy information about studies with human participants or human data. See also policy information about sex, gender (identity/presentation), and sexual orientation and race, ethnicity and racism.

Reporting on sex and gender	Not applicable
Reporting on race, ethnicity, or other socially relevant groupings	Not applicable
Population characteristics	Not applicable
Recruitment	Not applicable
Ethics oversight	Not applicable

Note that full information on the approval of the study protocol must also be provided in the manuscript.

Field-specific reporting

Please select the one below that is the best fit for your research. If you are not sure, read the appropriate sections before making your selection.

☒ Life sciences ☐ Behavioural & social sciences ☐ Ecological, evolutionary & environmental sciences

For a reference copy of the document with all sections, see nature.com/documents/nr-reporting-summary-flat.pdf

Life sciences study design

All studies must disclose on these points even when the disclosure is negative.

Sample size	Fifteen mice were used to observe survival. For experiments collecting independent values, we used 3-5 distinct samples for each group. These are based on our prior experience to acquire statistical significance. No sample size calculation was used.
Data exclusions	No data were excluded from analysis
Replication	To acquire independent values from distinct samples, three replications were used to generate one independent value for cell counting, qPCR, luciferase reporter assay and CCK-8.
Randomization	Mice (6 - 8 w, 18 - 20 g) were randomized prior to allocation to each group.
Blinding	Investigators were blinded in flow cytometry analysis and histological detections.

Reporting for specific materials, systems and methods

We require information from authors about some types of materials, experimental systems and methods used in many studies. Here, indicate whether each material, system or method listed is relevant to your study. If you are not sure if a list item applies to your research, read the appropriate section before selecting a response.

Materials & experimental systems

n/a	Involved in the study	
☐	☒	Antibodies
☐	☒	Eukaryotic cell lines
☒	☐	Palaeontology and archaeology
☐	☒	Animals and other organisms
☒	☐	Clinical data
☒	☐	Dual use research of concern
☒	☐	Plants

Methods

n/a	Involved in the study	
☒	☐	ChIP-seq
☐	☒	Flow cytometry
☒	☐	MRI-based neuroimaging

Antibodies

Antibodies used	CD45, clone name: 30-F11, catalog No.: 103132, BioLegend; CD3e, clone name: 145-2C11, catalog No.: 100328, BioLegend; CD4, clone name: RM4-5, catalog No.: 100510, BioLegend; CD8a, clone name: 53-6.7, catalog No.: 100708, BioLegend; CD62L, clone name: MEL-14, catalog No.: 104435, BioLegend; EpCAM, clone name: G8.8, catalog No.: 563478, BD Biosciences; CD44, clone name: IM7,

	catalog No.: 17-0441-83, Thermo Fisher Scientific; STAT3, clone name: 79D7, catalog No.: 4904, Cell Signaling Technology; p-STAT3, clone name: D3A7, catalog No.: 9145, Cell Signaling Technology; Rabbit IgG, catalog No.: 2729, Cell Signaling Technology; IL-22RA1, catalog No.: 13462-1-AP, Proteintech; β-actin, catalog No.: 20536-1-AP, Proteintech; Collagen, catalog No.: 14695-1-AP, Proteintech; AHR, catalog No.: 28727-1-AP, Proteintech; Rabbit IgG, catalog No.: 30000-0-AP, Proteintech; AHR, clone name: 2D1F9, catalog No.: 67785-1-Ig, Proteintech; CoraLite488-conjugated Goat Anti-Rabbit IgG, catalog No.: SA00013-2, Proteintech.
Validation	To validate anti-AHR (28727-1-AP) for immunoprecipitation, we used the precipitate to detect ARNT protein which is a constitutive binding partner of AHR, and ARNT was detectable. To validate anti-AHR (28727-1-AP) for ChIP, we used the precipitate to detect DNA fragment of promotor area of CYP1A1, which is a reporter gene of AHR activation. The other antibodies were validated by the manufacturers.

Eukaryotic cell lines

Policy information about cell lines and Sex and Gender in Research

Cell line source(s)	The murine thymic epithelial cell line mTEC1 (derived from BALB/c mice) was kindly provided by Prof. Yu Zhang (Peking University, Beijing).
Authentication	The cells were not authenticated.
Mycoplasma contamination	The cells were tested negative for mycoplasma contamination.
Commonly misidentified lines (See ICLAC register)	No commonly misidentified lines were used in the study.

Animals and other research organisms

Policy information about studies involving animals; ARRIVE guidelines recommended for reporting animal research, and Sex and Gender in Research

Laboratory animals	Wild type C57BL/6 and BALB/c mice (6 - 8 w, 18 - 20 g) were used . C57BL/6 background genetically modified mice were used .
Wild animals	The study did not involve wild animals.
Reporting on sex	Sex was not considered in the study design. The proportions of male and female animals were similar in each group.
Field-collected samples	The study did not involve samples collected from the field.
Ethics oversight	Procedures regarding animal care and experiments were approved by Medical Ethics Committee of Xuzhou Medical University.

Note that full information on the approval of the study protocol must also be provided in the manuscript.

Flow Cytometry

Plots

Confirm that:

☒ The axis labels state the marker and fluorochrome used (e.g. CD4-FITC).

☒ The axis scales are clearly visible. Include numbers along axes only for bottom left plot of group (a 'group' is an analysis of identical markers).

☒ All plots are contour plots with outliers or pseudocolor plots.

☒ A numerical value for number of cells or percentage (with statistics) is provided.

Methodology

Sample preparation	To obtain single cell suspension of thymus cells, freshly dissected thymii were cut into small pieces and treated by digestion with collagenase D, DNase I and Dispase. The dispersed cells were re-suspended in RPMI-1640 medium containing 2% FBS and 5 mM EDTA. Blood samples were collected from mice orbital vein with EDTA as anti-coagulant.
Instrument	Cells were acquired on an LSRFortessa flow cytometer (BD Biosciences).
Software	Data were analyzed using FlowJo 7.6 software.
Cell population abundance	Sorting was not performed in this study.

Gating strategy

Thymocytes were gated on CD45+ cells, and were further analyzed for CD4 and CD8 subsets. CD4+ T cells and CD8+ T cells were gated on CD3+CD4+CD8- and CD3+CD8+CD4- subpopulations, and were further analyzed for expressions of CD44 and CD62L.

☒ Tick this box to confirm that a figure exemplifying the gating strategy is provided in the Supplementary Information.
